# Geophysical measurement for estimation of groundwater hydraulic properties

**DOI:** 10.1016/j.dib.2018.10.057

**Published:** 2018-10-25

**Authors:** Safaa F. Yasir, Janmaizatulriah Jani, Mazidah Mukri

**Affiliations:** Faculty of Civil Engineering, Universiti Technology Mara, UiTM, Shah Alam 40450, Malaysia

**Keywords:** Electrical resistivity imaging, Regression equation, Hydraulic conductivity

## Abstract

In the study, a relationship was establishment between electrical resistivity by using electrical resistivity imaging (ERI) technique with hydraulic conductivity. By using Schlumberger array configuration, 2D electrical resistivity image was produced by using ABEM SAS 4000 with eighty-one (81) electrodes (Loke, 2004) [1]. By using regression equation, hydraulic conductivity was calculated from electrical resistivity and this result was compared with the hydraulic conductivity obtained from pumping tests (Butler, 2005). This data suggested that electrical resistivity survey can be used as preliminary tool to assess any subsurface zone with non- invasive nondestructive for soil, reducing time and cost.

**Specifications table**

**Table:** 

Subject area	*Geo-Physics, Hydrology*				
More specific subject area	*Electrical Resistivity Imaging and Hydraulic conductivity*				
Type of data	*Image, text file*				
How data was acquired	*ABEM SAS 4000 equipment produce Electrical Resistivity Imaging Raw data*				
Data format	*Raw, analyzed*				
Experimental factors	*Raw data analyzed by RES2DINV software.*				
Experimental features	*Schlumberger configurations was used with eighty-one (81) numbers of electrode and four (4) resistivity land cables each one is 100 m. The electrode was connected to resistivity land cables using eighty-two (82) numbers of jumper cable. The total line of electrical resistivity survey line is 400 m.*				
Data source location	Line	First point		Last point	
		N	E	N	E
	Line 1	02° 35′ 58.1′′	102° 34′ 14.0"	02° 35′ 56.1′′	102° 34′ 26.6′′
	Line 2	02° 35′ 58.1′′	102° 34′ 14.0′′	02° 36′ 10.6′′	102° 34′12.3′′
Data accessibility	*Data is with this article*				
Related research article	[2], [3], [4]				

**Value of the data**

• The development of subsurface imaging techniques for IP investigations [5] may permit detailed characterization of the spatial variability of these hydraulic properties, with obvious potential for hydrological model parameterization.
• Estimates values of conductivity by this approach, can reduce uncertainties in numerical model calibration and improve data coverage, reducing time and cost of a hydrogeological investigation at a regional scale.
• This data can be used for relating the hydrogeological investigations to the hydraulic properties of aquifer such as hydraulic conductivity, transmissivity and storativity.

## 1 Data

**Fig. 1:**
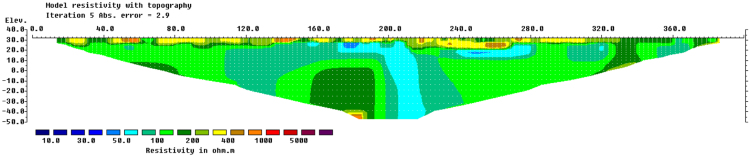
Resistivity image of Line one at Kg Bangkahulu, Gemas.

**Fig. 2:**
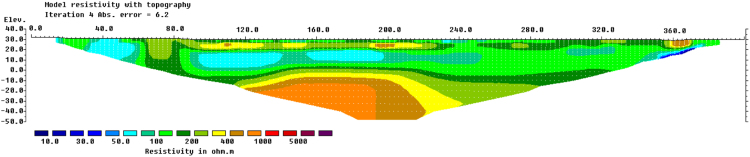
Resistivity image of Line two at Kg Bangkahulu, Gemas.

The Terrameter SAS 4000 data logger automatically saved the raw data from field 2D electrical resistivity imaging and this data was transformed to the computer for processing. For good understanding to the subsurface, a smooth boundary produced by using smooth constraint least square method. According to [6], this method is more suitable than a robust method for subsurface in contrast with fractured material which is sharp geomaterials boundary. The resistivity data was processed with RES2DINV software which is a computer program that will automatically determine a two-dimensional (2D) resistivity model for the subsurface for the data obtained. The inverse model resistivity sections are shown in Fig. 1 (line one) and Fig. 2 (line two).

## 2 Experimental design, materials, and methods

### 2.1 Analytical calculations using regression equation

In this paper, only line one selected for regression analyzing because the saturated zone much deeper and clearly appear in resistivity image and the tube well was drilling in the middle of this line. The raw data of resistivity was converted and saved to (XYZ) format by using RES2DINV Software to calculate the value of resistivity with different elevations from depth. The *K*-value was assumed depend on Bouwer׳s standard for hydraulic conductivity. Regression analysis is a statistical method used to formulate a mathematical equation in which the effect of one variable can be measured on the other where the purpose of using the simple linear regression method is to study and analysis the effect of a quantitative variable (hydraulic conductivity *K* regression) on another quantitative variable (resistivity) [7]. Regression equation is:

(1) $$\bar{y}=a+\mathrm{bX}$$

where Regression Coefficient is

(2) $$b=\frac{n\sum XY-\sum (X)\sum (Y)}{n\sum X^{2}-{(\sum X)}^{2}}$$

Gradient constant (fraction of the vertical axis *Y*) is

(3) $$a=\frac{\sum Y-b\sum X}{n}$$

where:

*X* = Resistivity in (Ohm-m), Ω
*Y* = Assumed hydraulic conductivity, (*K*-value)
$\bar{y}$ = Hydraulic conductivity (*K*_regression_) calculated from regression equation
*n* = number of the data for each variable

### 2.2 Tube well analyzing

*K*-value (hydraulic conductivity), used in all equations for groundwater flow. By using Darcy׳s law and continuity equation, *K*-value was calculated from pumping tests (drawdown, recovery, constant discharge).

(4) Q=V*A

(5) $$V=\frac{\Delta h}{T}$$

(6) $$K=\frac{Q*L}{A*\Delta h}$$

where: *Q* = Flow rate of pumping, *V* = The velocity of fluid, *A* = Area, Δh = The drawdown, *T* = Time, *L* = Length.
