# Supplementary material for: Geophysical measurement for estimation of groundwater hydraulic properties
Source: Data Brief. 2018 Oct 25;21:907–10. doi: 10.1016/j.dib.2018.10.057 (PMC6222259; doi:10.1016/j.dib.2018.10.057)
Supplement: Supplementary file 1 — Supplementary material [file mmc1.docx]

The drawdown test was conduct in five (5) phases as shown in Figure 1. The flow rate was (Q = 6.53 *m*^3^/*h*), (Q = 9.123 *m*^3^/*h*), (Q = 12.247 *m*^3^/*h*), (Q = 15.938 *m*^3^/*h*), (Q = 20.226 *m*^3^/*h*) for phase one, two, three, four, five respectively.


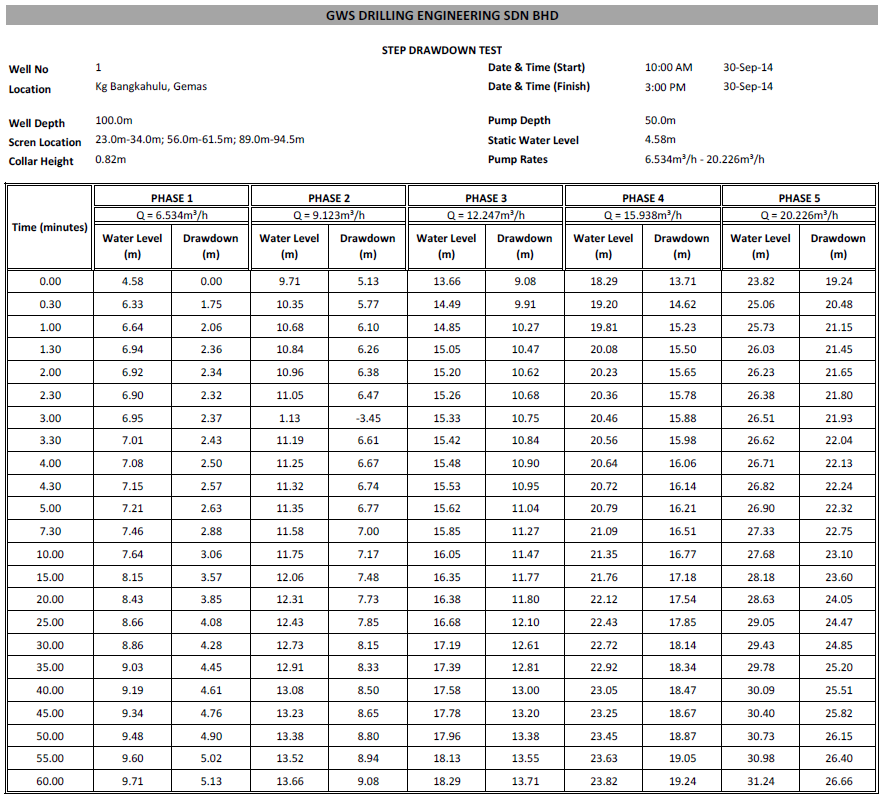


Figure 1 Step drawdown test with five phases.

The recovery test was conducted with rate of pumping (12.247 m^3^/h) for 20 hours after started of pumping and 8.3 hours after end of pumping. Figure 2, show the recovery test for Kg. Bangkahulu, Gemas site.


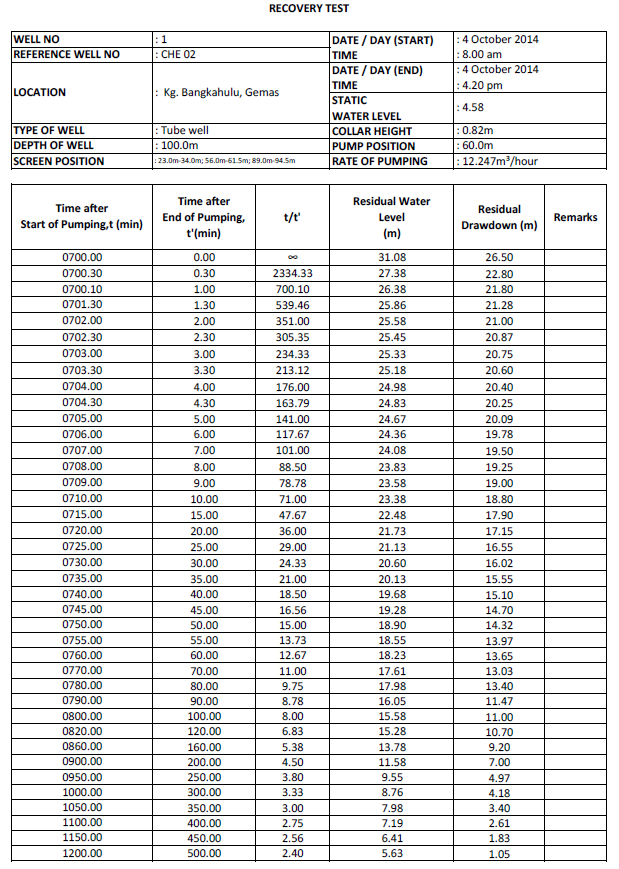


Figure 2 Recovery test for Kg. Bangkahulu, Gemas.

Figure 3 show the constant discharge test for Kg. Bangkahulu, Gemas.


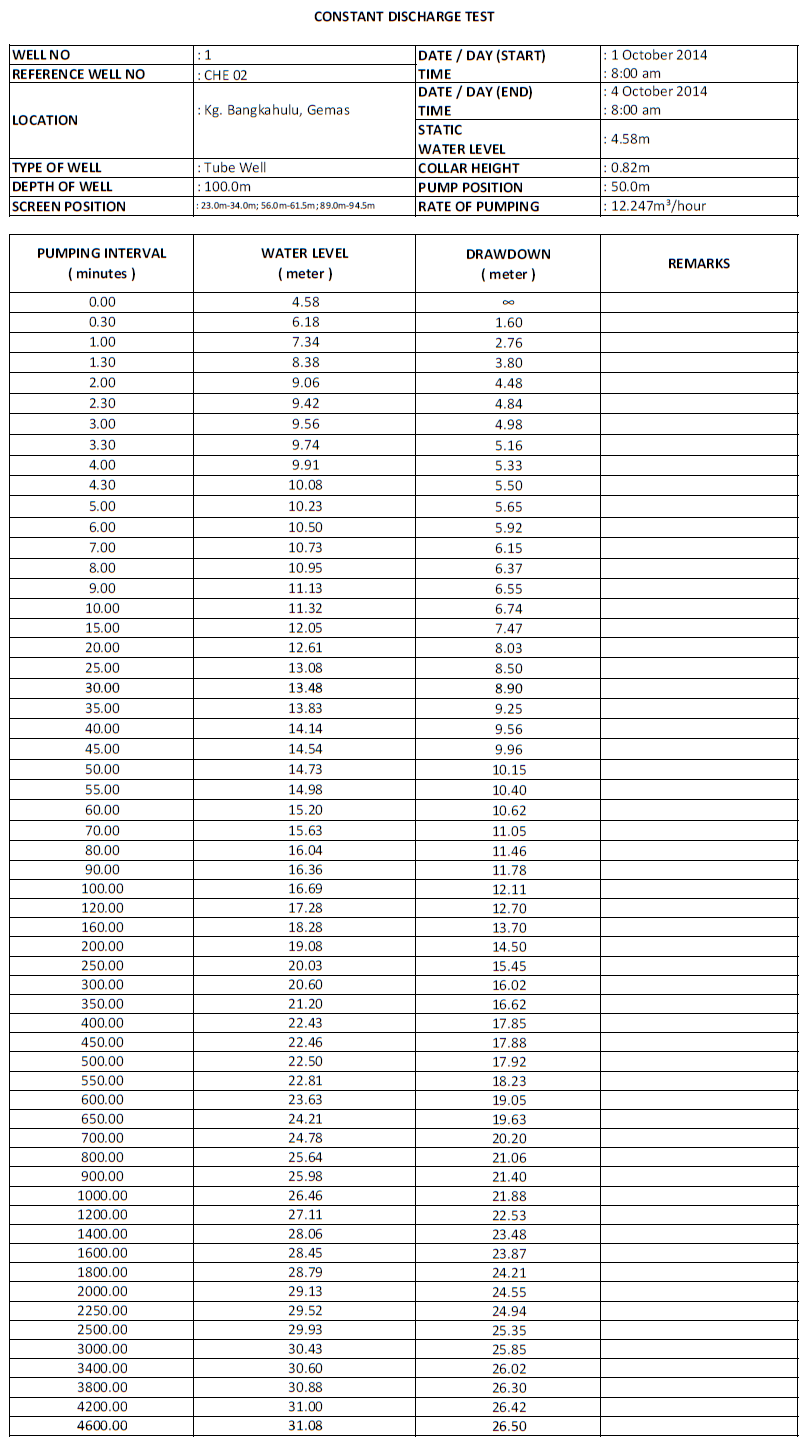


Figure 3 Constant Discharge Test for Kg. Bangkahulu, Gemas site.
